# Supplementary material for: The Evolution and Characterization of the RNA Interference Pathways in Lophotrochozoa
Source: Genome Biol Evol. 2024 May 7;16(5):evae098. doi: 10.1093/gbe/evae098 (PMC11114477; doi:10.1093/gbe/evae098)
Supplement: evae098_Supplementary_Data [file evae098_supplementary_data.zip › Supplementary_materials.zip!/selected_miRNAs/NoGe_selected_miRNAs/mirdeep_pdf_files/NMRB01000494.1_Notospermus_geniculatus_strain_Ushimado_scaffold494,_whole_genome_shotgun_sequence_14536.pdf]

Provisional ID : NMRB01000494.1_Notospermus_geniculatus_strain_Ushimado_scaffold494,_whole_genome_shotgun_sequence_14536
Score total : 111586.6
Score for star read(s) : 3.9
Score for read counts : 111577.9
Score for mfe : 1.8
Score for randfold : 0
Score for cons. seed : 3
Total read count : 218867
Mature read count : 218592
Loop read count : 0
Star read count : 275

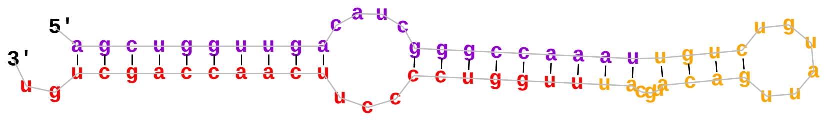

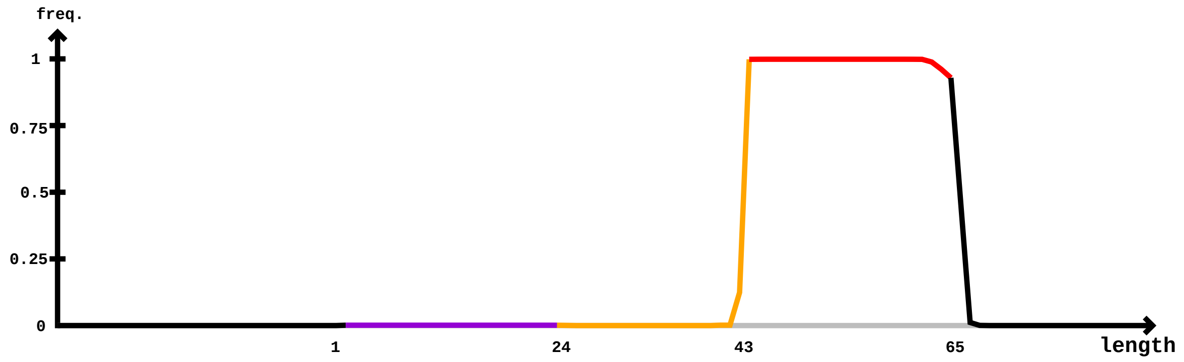

Star Mature

```
5'- aggaagcaaggaaaaaugcgauuucuguagcugguugacaucgggccaaauugucuguauugacaugcauuugguccccuucaaccagcuguagucagcauuacauacaaca   -3'   obs
    aggaagcaaggaaaaaugcgauuucuguagcugguugacaucgggccaaauugucuguauugacaugcauuugguccccuucaaccagcuguagucagcauuacauacaaca         exp
    ..............(((((.....((((((((((((((....(((((((((((((......))))...)))))))))...))))))))))))))...)))))..........   reads   mm   sample
    ............................agcugguugacaucgggccaaa..............................................................   1       0    Mm3
    ............................agcugguugaUaucgggccaaau.............................................................   1       1    Mm3
    ............................agcugguugacaucgggccaaau.............................................................   27      0    Mm3
    ............................agcugguugacaucggAccaaauu............................................................   1       1    Mm3
    ...................................................................cauuugguccccuucaaccagc.......................   17      0    Mm3
    ...................................................................cauuugguccccuucaCccagc.......................   1       1    Mm3
    ...................................................................cauuugguccccuucaaccagcu......................   2       0    Mm3
    ...................................................................cauuugguccccuucaaccagcuU.....................   1       1    Mm3
    .....................................................................uuugguccccuucaaccag........................   2       0    Mm3
    .....................................................................uuugguccccuucaaccagc.......................   17      0    Mm3
    .....................................................................uuugguccccuuUaaccagcu......................   1       1    Mm3
    .....................................................................uuugguccccuucaaccagcu......................   118     0    Mm3
    .....................................................................uuugguccccuucaaccUgcu......................   1       1    Mm3
    .....................................................................uuugguccccuucaaccagcG......................   2       1    Mm3
    .....................................................................uuugguccccAucaaccagcu......................   1       1    Mm3
    .....................................................................uuuggCccccuucaaccagcug.....................   1       1    Mm3
    .....................................................................uuugguccccuucaaccagcGg.....................   2       1    Mm3
    .....................................................................uuugguccccuucaaccagcug.....................   256     0    Mm3
    .....................................................................uuugguccccuucaaccGgcug.....................   1       1    Mm3
    .....................................................................uuugguccccuucaaccCgcug.....................   1       1    Mm3
    .....................................................................uuugguccccuucaacUagcug.....................   2       1    Mm3
    .....................................................................uuugguccccCucaaccagcug.....................   1       1    Mm3
    .....................................................................uuugguccccuucaaccUgcug.....................   1       1    Mm3
    .....................................................................uuugguccccuucaaccagcuCu....................   1       1    Mm3
    .....................................................................uuuAguccccuucaaccagcugu....................   1       1    Mm3
    .....................................................................uuugguccccuucaaccagcugC....................   4       1    Mm3
    .....................................................................uuugguccccuuUaaccagcugu....................   1       1    Mm3
    .....................................................................uuugguccccuucaacAagcugu....................   9       1    Mm3
    .....................................................................Nuugguccccuucaaccagcugu....................   1       1    Mm3
    .....................................................................uuugguccccuucaaccagcugu....................   2383    0    Mm3
    .....................................................................uuugguccccuucaaccCgcugu....................   5       1    Mm3
    .....................................................................uuugguccccuucaaccagcugA....................   8       1    Mm3
    .....................................................................uuugguccccuucaaccagcugG....................   5       1    Mm3
```

```
                            Star                                      Mature
aggaagcaaggaaaaaugcgauuucuguagcugguugacaucgggccaaauugucuguauugacaugcauuugguccccuucaaccagcuguagucagcauuacauacaaca
.....................................................................uuugguccccuucaaccGgcugu....................   8     1    Mm3
.....................................................................uuugguccccuucaacUagcugu....................   7     1    Mm3
.....................................................................uuugguccccuucaaccagcuUu....................   1     1    Mm3
.....................................................................uuugguccccCucaaccagcugu....................   4     1    Mm3
.....................................................................uuugguccccuucaaUcagcugu....................   7     1    Mm3
.....................................................................uuugguccccuAcaaccagcugu....................   1     1    Mm3
.....................................................................uuugguUcccuucaaccagcugu....................   1     1    Mm3
.....................................................................uuuggucUccuucaaccagcugu....................   1     1    Mm3
.....................................................................uuuggCccccuucaaccagcugu....................   2     1    Mm3
.....................................................................uuugguccccuucaaccUgcugu....................   5     1    Mm3
.....................................................................uuugguccccuucaacGagcugu....................   3     1    Mm3
.....................................................................uuugguccccAucaaccagcugu....................   1     1    Mm3
.....................................................................uAugguccccuucaaccagcugu....................   1     1    Mm3
.....................................................................uuugguccAcuucaaccagcugu....................   1     1    Mm3
.....................................................................uuuggucccUuucaaccagcugu....................   2     1    Mm3
.....................................................................Cuugguccccuucaaccagcugu....................   4     1    Mm3
.....................................................................uuugguAcccuucaaccagcugu....................   1     1    Mm3
.....................................................................uuugguccccuucaaccagcugua...................   49    0    Mm3
.....................................................................uuugguccccuucaaccagcuguC...................   1     1    Mm3
.....................................................................uuugguccccuucaaccagcugAa...................   1     1    Mm3
.....................................................................uuugguccccuucaaccagcuguU...................   439   1    Mm3
.....................................................................uuugguccccuucaaccagcuguaU..................   5     1    Mm3
.....................................................................uuugguccccuucaaccagcuguaA..................   1     1    Mm3
......................................................................uuggAccccuucaaccagc.......................   1     1    Mm3
......................................................................uugguccccuucaaccUgc.......................   1     1    Mm3
......................................................................uugguccccuucaaccagc.......................   70    0    Mm3
......................................................................uugguccccuucaaccagU.......................   1     1    Mm3
......................................................................uugguccccuucaaccagcA......................   1     1    Mm3
......................................................................uuggucccUuucaaccagcu......................   1     1    Mm3
......................................................................uugguccccuucaaccagcu......................   258   0    Mm3
......................................................................uugguccccuucaaccCgcu......................   2     1    Mm3
......................................................................uugguccccuucaaccagcG......................   5     1    Mm3
......................................................................uugguccccuAcaaccagcu......................   2     1    Mm3
......................................................................uugguccccuuUaaccagcu......................   1     1    Mm3
......................................................................uugguccccCucaaccagcu......................   1     1    Mm3
......................................................................uugguccccuucaaccaUcu......................   2     1    Mm3
......................................................................uugguccccuucaaccagcuU.....................   13    1    Mm3
......................................................................uugguccccuucaaccaCcug.....................   1     1    Mm3
......................................................................uugguccccuucaaccGgcug.....................   1     1    Mm3
......................................................................uugguccccuucaaccaAcug.....................   2     1    Mm3
......................................................................uugguccccuuUaaccagcug.....................   1     1    Mm3
......................................................................uugguccccuucaaccUgcug.....................   2     1    Mm3
......................................................................uugguccccuucaaUcagcug.....................   2     1    Mm3
......................................................................uugguccccuucaaccaUcug.....................   2     1    Mm3
......................................................................uugguccccuucaaccCgcug.....................   1     1    Mm3
......................................................................uugguccccuucaaccagcug.....................   369   0    Mm3
......................................................................uugguccccuucaacUagcug.....................   1     1    Mm3
......................................................................uugguccccuucUaccagcugu....................   2     1    Mm3
......................................................................uugguccccuucaaccagcAgu....................   2     1    Mm3
......................................................................uugguccccuuAaaccagcugu....................   1     1    Mm3
......................................................................uugguccccuucaaccagcugA....................   33    1    Mm3
......................................................................uuCguccccuucaaccagcugu....................   1     1    Mm3
......................................................................uugguccccuucaUccagcugu....................   1     1    Mm3
......................................................................uugguccccuucaacUagcugu....................   55    1    Mm3
......................................................................uugguccccuucaaccagAugu....................   2     1    Mm3
......................................................................uugguccccuucaaccagcugG....................   27    1    Mm3
......................................................................uCgguccccuucaaccagcugu....................   1     1    Mm3
......................................................................uuggucUccuucaaccagcugu....................   5     1    Mm3
......................................................................uugguccccuucaaccaUcugu....................   79    1    Mm3
......................................................................uugguccccuucaaccagUugu....................   5     1    Mm3
......................................................................uugguccccuucaCccagcugu....................   2     1    Mm3
......................................................................uugguccccuucaacAagcugu....................   1     1    Mm3
......................................................................uugguccUcuucaaccagcugu....................   4     1    Mm3
......................................................................uugguccccuucaaccGgcugu....................   16    1    Mm3
......................................................................uugguccccuucaaUcagcugu....................   18    1    Mm3
......................................................................uugguccccuucaaccaCcugu....................   59    1    Mm3
......................................................................uuUguccccuucaaccagcugu....................   3     1    Mm3
......................................................................uugUuccccuucaaccagcugu....................   2     1    Mm3
......................................................................Augguccccuucaaccagcugu....................   2     1    Mm3
......................................................................uugAuccccuucaaccagcugu....................   5     1    Mm3
```

Star Mature

```
aggaagcaaggaaaaaugcgauuucuguagcugguugacaucgggccaaauugucuguauugacaugcauuugguccccuucaaccagcuguagucagcauuacauacaaca
......................................................................uuggucGcuucaaccagcugu....................   1       1   Mm3
......................................................................uugguUcccuucaaccagcugu....................   8       1   Mm3
......................................................................uugguccccuucaaccagcuCu....................   1       1   Mm3
......................................................................Cugguccccuucaaccagcugu....................   4       1   Mm3
......................................................................uuggAccccuucaaccagcugu....................   1       1   Mm3
......................................................................uugguccccuucaaccagcugu....................   13980   0   Mm3
......................................................................uugguccccAucaaccagcugu....................   5       1   Mm3
......................................................................uugguccccuucaaccUgcugu....................   19      1   Mm3
......................................................................uGgguccccuucaaccagcugu....................   1       1   Mm3
......................................................................uuggucAccuucaaccagcugu....................   1       1   Mm3
......................................................................uuggucccUuucaaccagcugu....................   8       1   Mm3
......................................................................uugguccccuucaaccagcugC....................   12      1   Mm3
......................................................................uugCuccccuucaaccagcugu....................   1       1   Mm3
......................................................................uugguccccuucaaAcagcugu....................   3       1   Mm3
......................................................................uuggCccccuucaaccagcugu....................   5       1   Mm3
......................................................................uugguccccCucaaccagcugu....................   3       1   Mm3
......................................................................uugguccccuuUaaccagcugu....................   8       1   Mm3
......................................................................uugguAcccuucaaccagcugu....................   2       1   Mm3
......................................................................uAgguccccuucaaccagcugu....................   5       1   Mm3
......................................................................uugguccccuucaaccaAcugu....................   67      1   Mm3
......................................................................Nugguccccuucaaccagcugu....................   13      1   Mm3
......................................................................uugguccccuGcaaccagcugu....................   25      1   Mm3
......................................................................uuggucccAuucaaccagcugu....................   3       1   Mm3
......................................................................uuggGccccuucaaccagcugu....................   2       1   Mm3
......................................................................uugguGcccuucaaccagcugu....................   2       1   Mm3
......................................................................uugguccccuAcaaccagcugu....................   25      1   Mm3
......................................................................uugguccccuucaaccagcuAu....................   3       1   Mm3
......................................................................uugguccccuCcaaccagcugu....................   18      1   Mm3
......................................................................uugguccccuucaaccagcuUu....................   2       1   Mm3
......................................................................uugguccccuucaGccagcugu....................   1       1   Mm3
......................................................................uugguccccuucaaccCgcugu....................   23      1   Mm3
......................................................................uuAguccccuucaaccagcugu....................   2       1   Mm3
......................................................................uugguccccuucaaccagcugCa...................   1       1   Mm3
......................................................................uugguccccuucaaccaCcugua...................   2       1   Mm3
......................................................................uugUuccccuucaaccagcugua...................   2       1   Mm3
......................................................................uugguccccuGcaaccagcugua...................   1       1   Mm3
......................................................................uugguccccuucaaccagcuguU...................   6650    1   Mm3
......................................................................uugguccccuucaaccagcugua...................   406     0   Mm3
......................................................................Cugguccccuucaaccagcugua...................   1       1   Mm3
......................................................................uugguccccuucaaccaAcugua...................   2       1   Mm3
......................................................................uugguccccuucaaccagUugua...................   1       1   Mm3
......................................................................uugguccccuucaaccagcugAa...................   1       1   Mm3
......................................................................uugguccccuucaacUagcugua...................   3       1   Mm3
......................................................................uugguccccuucaaccagcuguG...................   4       1   Mm3
......................................................................uugguccccuucaaccaUcugua...................   1       1   Mm3
......................................................................uugguccccuCcaaccagcugua...................   1       1   Mm3
......................................................................uugguccccuucaaccagcuUua...................   1       1   Mm3
......................................................................uugguUcccuucaaccagcugua...................   1       1   Mm3
......................................................................uugguccccuucaaccagcuguC...................   11      1   Mm3
......................................................................uugguccccuucaaccagcuguaA..................   55      1   Mm3
......................................................................uugguccccuucaaccagcuguag..................   1       0   Mm3
......................................................................uugguccccuucaaccagcuguaC..................   2       1   Mm3
......................................................................uugguccccuucaaccagcuguaU..................   147     1   Mm3
......................................................................uugguccccuucaaccagcuguUg..................   11      1   Mm3
......................................................................uugguccccuucaaccagcuguaAu.................   5       1   Mm3
......................................................................uugguccccuucaaccagcuguUgu.................   2       1   Mm3
......................................................................uugguccccuucaaccagcuguaUu.................   14      1   Mm3
.......................................................................ugguccccuuUaaccagcugu....................   1       1   Mm3
.......................................................................ugguccccuucaaccagcugu....................   7       0   Mm3
.......................................................................ugAuccccuucaaccagcugu....................   1       1   Mm3
.......................................................................ugguccccuucaaccagcugua...................   2       0   Mm3
.......................................................................ugguccccuucaaccagcuguU...................   9       1   Mm3
........................................................................gguccccuucaaccagcugu....................   1       0   Mm3
..........................................................................uccccuucaaccagcugu....................   1       0   Mm3

............................agcugguugacaucgggcc.................................................................   1       0   Fm3
............................agcugguugacaucgggcca................................................................   3       0   Fm3
............................agcugguugacaucgggccaa...............................................................   1       0   Fm3
............................agcugguugacaucgggccaaa..............................................................   5       0   Fm3
............................agcugguugacaucgggUcaaau.............................................................   2       1   Fm3
```

```
                           Star                                        Mature
aggaagcaaggaaaaaugcgauuucuguagcugguugacaucgggccaaauugucuguauugacaugcauuugguccccuucaaccagcuguagucagcauuacauacaaca
............................agcugguugacaucgggGcaaau.............................................................   1     1    Fm3
............................agcugguugacaucgggccaaau.............................................................   120   0    Fm3
............................agcugguugaAaucgggccaaau.............................................................   1     1    Fm3
............................agcugguugacaucgggAcaaau.............................................................   1     1    Fm3
...................................................................cauuugguccccuucaaccag........................   3     0    Fm3
...................................................................cauuugguccccuucaaAcagc.......................   2     1    Fm3
...................................................................cauuugguccccuucaaccagA.......................   3     1    Fm3
...................................................................cauuugguccccuucaaccagc.......................   90    0    Fm3
...................................................................cauuugguccccuucaaGcagc.......................   1     1    Fm3
...................................................................cauuugguccccuucaaccagcu......................   6     0    Fm3
....................................................................Cuuugguccccuucaaccagcug.....................   1     1    Fm3
.....................................................................Nuugguccccuucaacca.........................   1     1    Fm3
.....................................................................uuugguccccuucaacca.........................   13    0    Fm3
.....................................................................uuugguccccuucaaccag........................   5     0    Fm3
.....................................................................uuugguccccuucaaccagc.......................   37    0    Fm3
.....................................................................uuuggucccUuucaaccagc.......................   1     1    Fm3
.....................................................................uuugguccccuucaacUagcu......................   2     1    Fm3
.....................................................................uuuUguccccuucaaccagcu......................   1     1    Fm3
.....................................................................uuugguccccuucaaccCgcu......................   1     1    Fm3
.....................................................................uuugguccccuucaaccagcu......................   99    0    Fm3
.....................................................................uuugguccccuucCaccagcug.....................   1     1    Fm3
.....................................................................uuugguccccuucaaccCgcug.....................   2     1    Fm3
.....................................................................uuugguccccuucaacAagcug.....................   1     1    Fm3
.....................................................................uuugguAcccuucaaccagcug.....................   1     1    Fm3
.....................................................................uuugguccccuucGaccagcug.....................   1     1    Fm3
.....................................................................uuugguccccuucaaUcagcug.....................   2     1    Fm3
.....................................................................uuugguccccuucaaccagcug.....................   277   0    Fm3
.....................................................................uuugguccccuuUaaccagcug.....................   1     1    Fm3
.....................................................................uuugguccccuucaacUagcug.....................   1     1    Fm3
.....................................................................uuugguccccAucaaccagcug.....................   1     1    Fm3
.....................................................................uuugguccccuucaaccagcuA.....................   1     1    Fm3
.....................................................................uuugguccccuucaaccUgcug.....................   1     1    Fm3
.....................................................................uuGgguccccuucaaccagcug.....................   1     1    Fm3
.....................................................................uuugguccUcuucaaccagcugu....................   1     1    Fm3
.....................................................................uuugguccccGucaaccagcugu....................   2     1    Fm3
.....................................................................uuugguccccuucaaccGgcugu....................   5     1    Fm3
.....................................................................uuugguccccuucaaccagcugA....................   7     1    Fm3
.....................................................................uuugguccccuucaaccagcugu....................   519   0    Fm3
.....................................................................uuuAguccccuucaaccagcugu....................   1     1    Fm3
.....................................................................uuuggucccUuucaaccagcugu....................   1     1    Fm3
.....................................................................uuugAuccccuucaaccagcugu....................   1     1    Fm3
.....................................................................uuugguccccuucaacUagcugu....................   4     1    Fm3
.....................................................................uuugguccccuucaaccCgcugu....................   2     1    Fm3
.....................................................................uuugguccccuucaacGagcugu....................   1     1    Fm3
.....................................................................uuugguccccuucaaUcagcugu....................   5     1    Fm3
.....................................................................uuugguccccuucaaccagUugu....................   2     1    Fm3
.....................................................................Nuugguccccuucaaccagcugu....................   1     1    Fm3
.....................................................................uuGgguccccuucaaccagcugu....................   1     1    Fm3
.....................................................................uuugguccccuucaacAagcugu....................   1     1    Fm3
.....................................................................uuugguccccuucaaccagcuguU...................   97    1    Fm3
.....................................................................uuugguccccuucaaccagcugua...................   17    0    Fm3
.....................................................................uuugguccccuucaaccagcuguaU..................   3     1    Fm3
.....................................................................uuugguccccuucaaccagcuguag..................   1     0    Fm3
......................................................................uugguccccuucGaccag........................   1     1    Fm3
......................................................................uugguccccuucaaccag........................   20    0    Fm3
......................................................................uugguccccuucaaccaAc.......................   1     1    Fm3
......................................................................Cugguccccuucaaccagc.......................   1     1    Fm3
......................................................................uugguccccuucaaUcagc.......................   2     1    Fm3
......................................................................uugguccccuucaaccUgc.......................   1     1    Fm3
......................................................................uugguccccuGcaaccagc.......................   1     1    Fm3
......................................................................uugguccccuucaaccaCc.......................   3     1    Fm3
......................................................................uugguccccuucaaccagA.......................   16    1    Fm3
......................................................................uugguccccuucaaccagU.......................   12    1    Fm3
......................................................................uuggucAccuucaaccagc.......................   1     1    Fm3
......................................................................uugguccccuucaaccagc.......................   331   0    Fm3
......................................................................uuggucUccuucaaccagc.......................   1     1    Fm3
......................................................................uuggucAccuucaaccagcu......................   1     1    Fm3
......................................................................uugguccccuucaaccagcu......................   525   0    Fm3
......................................................................uugguccccuucaaccGgcu......................   1     1    Fm3
......................................................................uugguccccuCcaaccagcu......................   1     1    Fm3
```

Star Mature

```
aggaagcaaggaaaaaugcgauuucuguagcugguugacaucgggccaaauugucuguauugacaugcauuugguccccuucaaccagcuguagucagcauuacauacaaca
......................................................................uugguccccuucaaccagUu......................  3     1  Fm3
......................................................................uugguccccuucaacUagcu......................  3     1  Fm3
......................................................................uugguccccuucaaccCgcu......................  1     1  Fm3
......................................................................uugguccccuucaaccaAcu......................  2     1  Fm3
......................................................................uugguccccuucaaccaUcu......................  2     1  Fm3
......................................................................uugguAcccuucaaccagcu......................  1     1  Fm3
......................................................................uugguccccuucaaccagcC......................  2     1  Fm3
......................................................................uugguccccuucaaccagcA......................  4     1  Fm3
......................................................................uugAuccccuucaaccagcu......................  1     1  Fm3
......................................................................uugguccccuucaaccaCcu......................  4     1  Fm3
......................................................................Nugguccccuucaaccagcu......................  1     1  Fm3
......................................................................uugguUcccuucaaccagcug.....................  1     1  Fm3
......................................................................uugguccccuucaaccaCcug.....................  5     1  Fm3
......................................................................uugguccccuucaacAagcug.....................  2     1  Fm3
......................................................................uugguccccuucaaccagcuA.....................  3     1  Fm3
......................................................................uuUguccccuucaaccagcug.....................  1     1  Fm3
......................................................................uugguccccuucaaccaAcug.....................  4     1  Fm3
......................................................................uugguccccuucaaccagcuU.....................  4     1  Fm3
......................................................................uugguccccuucaaUcagcug.....................  4     1  Fm3
......................................................................uuggucccAuucaaccagcug.....................  1     1  Fm3
......................................................................uGgguccccuucaaccagcug.....................  1     1  Fm3
......................................................................uugguccccuucaaccagUug.....................  1     1  Fm3
......................................................................uugguccccuAcaaccagcug.....................  3     1  Fm3
......................................................................uugguccccuucaaccagcug.....................  693   0  Fm3
......................................................................uugguAcccuucaaccagcug.....................  1     1  Fm3
......................................................................uugguccccuucaaccUgcug.....................  1     1  Fm3
......................................................................uugguccccuucaUccagcug.....................  1     1  Fm3
......................................................................uugguccccuucaaccaUcug.....................  6     1  Fm3
......................................................................uugguccccuGcaaccagcug.....................  2     1  Fm3
......................................................................uGgguccccuucaaccagcugu....................  1     1  Fm3
......................................................................uuggucccAuucaaccagcugu....................  3     1  Fm3
......................................................................uugAuccccuucaaccagcugu....................  1     1  Fm3
......................................................................uugguccccuucaaccagcugu....................  7111  0  Fm3
......................................................................uugguccccuucaaccagcAgu....................  1     1  Fm3
......................................................................uugguNcccuucaaccagcugu....................  2     1  Fm3
......................................................................uuggCccccuucaaccagcugu....................  3     1  Fm3
......................................................................uugguccccuucaaccCgcugu....................  19    1  Fm3
......................................................................uugguccccuucUaccagcugu....................  2     1  Fm3
......................................................................uugguccccAucaaccagcugu....................  1     1  Fm3
......................................................................uuggGccccuucaaccagcugu....................  2     1  Fm3
......................................................................uuggucGccuucaaccagcugu....................  2     1  Fm3
......................................................................uugguccccuucaaccagcCgu....................  1     1  Fm3
......................................................................uAgguccccuucaaccagcugu....................  2     1  Fm3
......................................................................uugguAcccuucaaccagcugu....................  1     1  Fm3
......................................................................uugguccccuucaacUagcugu....................  62    1  Fm3
......................................................................uuggucccUuucaaccagcugu....................  12    1  Fm3
......................................................................uugguccccCucaaccagcugu....................  3     1  Fm3
......................................................................uugguccccuucaaccagcuAu....................  2     1  Fm3
......................................................................uugguccccuucaaccaAcugu....................  31    1  Fm3
......................................................................uugguccccuCcaaccagcugu....................  4     1  Fm3
......................................................................uugguccccuucaaccagGugu....................  1     1  Fm3
......................................................................uugguccccuAcaaccagcugu....................  14    1  Fm3
......................................................................uuggucUccuucaaccagcugu....................  3     1  Fm3
......................................................................uugguccccuucaaccagcuUu....................  3     1  Fm3
......................................................................uuggAccccuucaaccagcugu....................  3     1  Fm3
......................................................................uugguccccuucaaccUgcugu....................  23    1  Fm3
......................................................................uugguccccuucaaccagcugA....................  40    1  Fm3
......................................................................Nugguccccuucaaccagcugu....................  3     1  Fm3
......................................................................uugguccccuucGaccagcugu....................  1     1  Fm3
......................................................................uugguccUcuucaaccagcugu....................  6     1  Fm3
......................................................................uugguccccuucaaccaCcugu....................  43    1  Fm3
......................................................................uuUguccccuucaaccagcugu....................  4     1  Fm3
......................................................................uugguccccuucaaccaUcugu....................  45    1  Fm3
......................................................................uugguccccuucaGccagcugu....................  1     1  Fm3
......................................................................uugguccccuucaaccGgcugu....................  2     1  Fm3
......................................................................uugguccccuGcaaccagcugu....................  13    1  Fm3
......................................................................uugguccccuucaUccagcugu....................  2     1  Fm3
......................................................................uugguccAcuucaaccagcugu....................  6     1  Fm3
......................................................................uugCuccccuucaaccagcugu....................  1     1  Fm3
......................................................................uugguccccuucaaccagcGgu....................  1     1  Fm3
```

```
                            Star                                       Mature
aggaagcaaggaaaaaugcgauuucuguagcugguugacaucgggccaaauugucuguauugacaugcauuugguccccuucaaccagcuguagucagcauuacauacaaca
......................................................................uugguccccuucaaccagUugu....................    2     1    Fm3
......................................................................uugguUcccuucaaccagcugu....................    1     1    Fm3
......................................................................uugguccccuucaacAagcugu....................    2     1    Fm3
......................................................................uCgguccccuucaaccagcugu....................    2     1    Fm3
......................................................................uugguccccuucaaUcagcugu....................    43    1    Fm3
......................................................................uugguccccuucaaccagcugC....................    2     1    Fm3
......................................................................uugguccccuuUaaccagcugu....................    7     1    Fm3
......................................................................uuggucAccuucaaccagcugu....................    3     1    Fm3
......................................................................uugguccccuucaaccagcuguU...................    2612  1    Fm3
......................................................................uugguccccuucaaccagcuguG...................    1     1    Fm3
......................................................................uugguccccuucaaccUgcugua...................    1     1    Fm3
......................................................................uugguccccuCcaaccagcugua...................    1     1    Fm3
......................................................................uugguccccuucaaccaAcugua...................    2     1    Fm3
......................................................................uugguccccuucaaccaUcugua...................    2     1    Fm3
......................................................................uugguccccuucaacUagcugua...................    3     1    Fm3
......................................................................uugguccccuAcaaccagcugua...................    2     1    Fm3
......................................................................uugguccccuucaaccagcugAa...................    2     1    Fm3
......................................................................uugguccccuucaaccCgcugua...................    1     1    Fm3
......................................................................uuggucUccuucaaccagcugua...................    1     1    Fm3
......................................................................uugguccccuucaaccagcuguC...................    9     1    Fm3
......................................................................uugguccccuucaaccaCcugua...................    1     1    Fm3
......................................................................uugguccccuucaaccagcuCua...................    1     1    Fm3
......................................................................Nugguccccuucaaccagcugua...................    1     1    Fm3
......................................................................uugguccccuucaaccagcugua...................    446   0    Fm3
......................................................................uugguccccuucaaccGgcugua...................    1     1    Fm3
......................................................................uugguccccuucaaccagcugCa...................    1     1    Fm3
......................................................................uugguccccuucaaccagcuguag..................    3     0    Fm3
......................................................................uugguccccuucaaccagcuguaA..................    64    1    Fm3
......................................................................uugguccccuucaaccagcuguaU..................    160   1    Fm3
......................................................................uugguccccuucaaccagcuguagA.................    1     1    Fm3
......................................................................uugguccccuucaaccagcuguaUu.................    6     1    Fm3
......................................................................uugguccccuucaaccagcuguaAu.................    3     1    Fm3
.......................................................................ugguccccuucaaccagc.......................    1     0    Fm3
.......................................................................ugguccccuucaaccagcugu....................    2     0    Fm3
.......................................................................uUguccccuucaaccagcugu....................    1     1    Fm3
.......................................................................ugguccccuucaaccagcugua...................    1     0    Fm3
..........................................................................uccccuucaaccagcuAu....................    1     1    Fm3
..........................................................................uccccuucaaccagcugu....................    2     0    Fm3

............................agcugguugacaucgggccaaau.............................................................    17    0    Mm1
............................agcugguugacaucgggccaaauugucug.......................................................    3     0    Mm1
...................................................................cauuugguccccuucaaccagc.......................    4     0    Mm1
...................................................................cauuugguccccuucaaccagcu......................    7     0    Mm1
...................................................................cauuugguccccuucaaGcagcu......................    1     1    Mm1
.....................................................................uuugguccccuucaaccagc.......................    21    0    Mm1
.....................................................................uuugguccccuucaaccGgcu......................    1     1    Mm1
.....................................................................uuugguccccuucaacAagcu......................    1     1    Mm1
.....................................................................uuugguccccuucaaccagcu......................    45    0    Mm1
.....................................................................uuugguccccuucaacUagcu......................    1     1    Mm1
.....................................................................uuugguccccuucaaUcagcug.....................    10    1    Mm1
.....................................................................uuugguccccuucaaccagcug.....................    84    0    Mm1
.....................................................................uuugguccccuucaaccUgcug.....................    1     1    Mm1
.....................................................................uuugguccccuucaaccGgcug.....................    1     1    Mm1
.....................................................................uuugguccAcuucaaccagcugu....................    1     1    Mm1
.....................................................................uuugguccccuucaaccCgcugu....................    8     1    Mm1
.....................................................................uuugguccccuucaaccagAugu....................    1     1    Mm1
.....................................................................uuugguccGcuucaaccagcugu....................    1     1    Mm1
.....................................................................uNugguccccuucaaccagcugu....................    1     1    Mm1
.....................................................................uuugguccccuucaacAagcugu....................    4     1    Mm1
.....................................................................uuugguccccuucaacGagcugu....................    2     1    Mm1
.....................................................................uuugguccccuucaacUagcugu....................    12    1    Mm1
.....................................................................uuugguccccGucaaccagcugu....................    1     1    Mm1
.....................................................................uuugguccccuucaaccagcugu....................    999   0    Mm1
.....................................................................uuugguccccuucaaccagcugA....................    11    1    Mm1
.....................................................................uuugguccccAucaaccagcugu....................    1     1    Mm1
.....................................................................uuugguccccuucaaccagcugG....................    1     1    Mm1
.....................................................................uuugguccccuucaaccUgcugu....................    4     1    Mm1
.....................................................................uuugguccccuucaaccGgcugu....................    2     1    Mm1
.....................................................................uuugguccccuucaaccagcugua...................    5     0    Mm1
.....................................................................uuugguccccuucaaccagcuguU...................    212   1    Mm1
```

```
                            Star                                      Mature
aggaagcaaggaaaaaugcgauuucuguagcugguugacaucgggccaaauugucuguauugacaugcauuugguccccuucaaccagcuguagucagcauuacauacaaca
......................................................................uugguccccuucaaccaCc.......................    1     1     Mm1
......................................................................uugguccccuucaaccaAc.......................    1     1     Mm1
......................................................................uuAguccccuucaaccagc.......................    1     1     Mm1
......................................................................uugguccccuucaaccagc.......................    91    0     Mm1
......................................................................uugguccccuAcaaccagc.......................    1     1     Mm1
......................................................................uugguccccuucaaccUgcu......................    1     1     Mm1
......................................................................uugguccccuucaaccaAcu......................    1     1     Mm1
......................................................................uugguccccAucaaccagcu......................    1     1     Mm1
......................................................................uugguccccuucaaccagcu......................    181   0     Mm1
......................................................................uugguccccuucGaccagcu......................    1     1     Mm1
......................................................................uugguccccuucaaccaCcug.....................    1     1     Mm1
......................................................................uugguccccuucaacUagcug.....................    8     1     Mm1
......................................................................uugguccccuucaaccagcuU.....................    10    1     Mm1
......................................................................uugguccccuucaaccagcug.....................    112   0     Mm1
......................................................................uuAguccccuucaaccagcugu....................    6     1     Mm1
......................................................................uuggAccccuucaaccagcugu....................    1     1     Mm1
......................................................................Cugguccccuucaaccagcugu....................    1     1     Mm1
......................................................................uuggucNccuucaaccagcugu....................    2     1     Mm1
......................................................................uugguccccCucaaccagcugu....................    1     1     Mm1
......................................................................Nugguccccuucaaccagcugu....................    6     1     Mm1
......................................................................uugAuccccuucaaccagcugu....................    2     1     Mm1
......................................................................uuUguccccuucaaccagcugu....................    2     1     Mm1
......................................................................uugguccccuucCaccagcugu....................    1     1     Mm1
......................................................................uGgguccccuucaaccagcugu....................    1     1     Mm1
......................................................................uugguccccuucaaccUgcugu....................    12    1     Mm1
......................................................................uuggucAccuucaaccagcugu....................    1     1     Mm1
......................................................................uAgguccccuucaaccagcugu....................    2     1     Mm1
......................................................................uugguccccuucGaccagcugu....................    3     1     Mm1
......................................................................uugguccccuucaaccaUcugu....................    27    1     Mm1
......................................................................uugguccccuucaaccaAcugu....................    30    1     Mm1
......................................................................uugguccccuuUaaccagcugu....................    2     1     Mm1
......................................................................uugguccccuucaaccaCcugu....................    40    1     Mm1
......................................................................uugguccccuucaaccagcuUu....................    6     1     Mm1
......................................................................uugguccccuucaaccagcugu....................    7076  0     Mm1
......................................................................uugguccccuCcaaccagcugu....................    4     1     Mm1
......................................................................uugguccccuucaaccagcAgu....................    1     1     Mm1
......................................................................uugguccccuucaaccagcugA....................    49    1     Mm1
......................................................................uuggCccccuucaaccagcugu....................    3     1     Mm1
......................................................................uugguccccGucaaccagcugu....................    1     1     Mm1
......................................................................uugguccccuucaacUagcugu....................    123   1     Mm1
......................................................................uugguccccuucaaccagAugu....................    1     1     Mm1
......................................................................uugguccccuucaaccagcugG....................    5     1     Mm1
......................................................................uugguccccuuGaaccagcugu....................    1     1     Mm1
......................................................................uugguUcccuucaaccagcugu....................    5     1     Mm1
......................................................................uuggucccUuucaaccagcugu....................    9     1     Mm1
......................................................................uugguccccuAcaaccagcugu....................    10    1     Mm1
......................................................................uugguccAcuucaaccagcugu....................    3     1     Mm1
......................................................................uugguccccuucaaccCgcugu....................    19    1     Mm1
......................................................................uuggucUccuucaaccagcugu....................    4     1     Mm1
......................................................................uugguccccuucaaccagcuAu....................    2     1     Mm1
......................................................................uugguccccuucaUccagcugu....................    1     1     Mm1
......................................................................uugguccccuucaaccGgcugu....................    9     1     Mm1
......................................................................uuggGccccuucaaccagcugu....................    2     1     Mm1
......................................................................uugguccccuucaaccagcCgu....................    2     1     Mm1
......................................................................uugguccccuucaaUcagcugu....................    11    1     Mm1
......................................................................uugguccccuGcaaccagcugu....................    9     1     Mm1
......................................................................uugguccccuucUaccagcugu....................    2     1     Mm1
......................................................................uugguccccuucaaccagcuguG...................    1     1     Mm1
......................................................................uugguccccuucaaccagcuAua...................    1     1     Mm1
......................................................................uugguccccuucaaccagcuguU...................    3985  1     Mm1
......................................................................uuggAccccuucaaccagcugua...................    1     1     Mm1
......................................................................uugguccccuucaaccagcuguC...................    16    1     Mm1
......................................................................uugguccccuucaaccGgcugua...................    1     1     Mm1
......................................................................uugguccccuucaaccaCcugua...................    1     1     Mm1
......................................................................uugguccccuucaaccUgcugua...................    1     1     Mm1
......................................................................uugguccccuucaaccagcugua...................    232   0     Mm1
......................................................................uugguccccuucaaccagcuguGg..................    1     1     Mm1
......................................................................uugguccccuucaaccagcuguaA..................    18    1     Mm1
......................................................................uugguccccuucaaccagcuguaU..................    67    1     Mm1
......................................................................uugguccccuucaaccagcuguag..................    5     0     Mm1
```

```
                           Star                                      Mature
aggaagcaaggaaaaaugcgauuucuguagcugguugacaucgggccaaauugucuguauugacaugcauuugguccccuucaaccagcuguagucagcauuacauacaaca
......................................................................uugguccccuucaaccagcuguaUu.................   5      1      Mm1
.......................................................................ugguccccuucaaccagcugu....................   2      0      Mm1
.......................................................................ugguccccuucaaccagcuguU...................   2      1      Mm1

............................agcugguugacaucgggccaaa..............................................................   1      0      Mm2
............................agcugguugacaucgggccaaau.............................................................   43     0      Mm2
............................agcugguugacaucggUccaaau.............................................................   1      1      Mm2
............................aUcugguugacaucgggccaaau.............................................................   1      1      Mm2
...................................................................cauuugguccccuucaaccU.........................   1      1      Mm2
...................................................................cauuugguccccuucaaccagc.......................   44     0      Mm2
...................................................................cauuugguccccuucaaccagcu......................   2      0      Mm2
....................................................................Cuuugguccccuucaaccagcugu....................   5      1      Mm2
.....................................................................uuugguccccuucaaccag........................   1      0      Mm2
.....................................................................uuugguccccuucaacUagc.......................   1      1      Mm2
.....................................................................uuugguccccuucaaccagc.......................   16     0      Mm2
.....................................................................uuugguccccuucaaccGgcu......................   2      1      Mm2
.....................................................................uuuggucccUuucaaccagcu......................   1      1      Mm2
.....................................................................uuugguNcccuucaaccagcu......................   1      1      Mm2
.....................................................................uuugguccccuucaaccagcu......................   122    0      Mm2
.....................................................................uuugguccccuucaaccagcG......................   5      1      Mm2
.....................................................................uuugguccccuucaaccUgcu......................   1      1      Mm2
.....................................................................uuugguccccuucaaccGgcug.....................   1      1      Mm2
.....................................................................uuugguccccuucaaccagcuU.....................   5      1      Mm2
.....................................................................uuugguccccuucaaccCgcug.....................   2      1      Mm2
.....................................................................uuugguccccuucaacGagcug.....................   1      1      Mm2
.....................................................................uuugguccccuucaaccagcug.....................   589    0      Mm2
.....................................................................uuugguccccuuUaaccagcug.....................   1      1      Mm2
.....................................................................uuugguccccuucaaccUgcug.....................   3      1      Mm2
.....................................................................uuugguccccuucaacUagcug.....................   2      1      Mm2
.....................................................................uAugguccccuucaaccagcug.....................   2      1      Mm2
.....................................................................uuugguccccuucaacAagcug.....................   3      1      Mm2
.....................................................................uuugguccccGucaaccagcug.....................   1      1      Mm2
.....................................................................uuugguccccuucaaccagcGg.....................   7      1      Mm2
.....................................................................uuGgguccccuucaaccagcug.....................   1      1      Mm2
.....................................................................uuugguccccuucaaccaAcugu....................   1      1      Mm2
.....................................................................uuugguccccuucaaccagUugu....................   1      1      Mm2
.....................................................................uuuggucccUuucaaccagcugu....................   3      1      Mm2
.....................................................................uuugguccccuucaacAagcugu....................   14     1      Mm2
.....................................................................uuugguccccuucaaccagcugG....................   9      1      Mm2
.....................................................................uuugguccccuucaaccagcugu....................   3594   0      Mm2
.....................................................................uuugguccccCucaaccagcugu....................   3      1      Mm2
.....................................................................uuugguccccuucaaccagcAgu....................   1      1      Mm2
.....................................................................Auugguccccuucaaccagcugu....................   1      1      Mm2
.....................................................................uuuCguccccuucaaccagcugu....................   1      1      Mm2
.....................................................................uuuggucUccuucaaccagcugu....................   2      1      Mm2
.....................................................................uuugguccAcuucaaccagcugu....................   1      1      Mm2
.....................................................................uuugguccccuucaaccagGugu....................   1      1      Mm2
.....................................................................uuugguccccuucaUccagcugu....................   1      1      Mm2
.....................................................................uuAgguccccuucaaccagcugu....................   2      1      Mm2
.....................................................................uuugguccccuucaacGagcugu....................   6      1      Mm2
.....................................................................Nuugguccccuucaaccagcugu....................   2      1      Mm2
.....................................................................uuugguccccGucaaccagcugu....................   5      1      Mm2
.....................................................................uuugguccccuCcaaccagcugu....................   1      1      Mm2
.....................................................................uuuUguccccuucaaccagcugu....................   1      1      Mm2
.....................................................................uuugguccccuucaaccagcugA....................   10     1      Mm2
.....................................................................uGugguccccuucaaccagcugu....................   1      1      Mm2
.....................................................................uuugguccccuucaaUcagcugu....................   3      1      Mm2
.....................................................................uuugguccccuucaaccCgcugu....................   11     1      Mm2
.....................................................................uuugguUcccuucaaccagcugu....................   1      1      Mm2
.....................................................................uAugguccccuucaaccagcugu....................   2      1      Mm2
.....................................................................uuugguccccuucaaccGgcugu....................   14     1      Mm2
.....................................................................uuuggCccccuucaaccagcugu....................   1      1      Mm2
.....................................................................uuugguccccuucaacUagcugu....................   19     1      Mm2
.....................................................................uuugguccccAucaaccagcugu....................   6      1      Mm2
.....................................................................uuugguccccuucaaccagcugC....................   6      1      Mm2
.....................................................................uuugguccccuucaaccUgcugu....................   8      1      Mm2
.....................................................................Cuugguccccuucaaccagcugu....................   7      1      Mm2
.....................................................................uuugguccccuuUaaccagcugu....................   3      1      Mm2
.....................................................................uuugguccccuucaaccaUcugu....................   1      1      Mm2
.....................................................................uuugguNcccuucaaccagcugu....................   1      1      Mm2
```

Star **Mature**

aggaagcaaggaaaaaugcgauuucuguagcugguugacaucgggccaaauugucuguauugacaugcauuugguccccuucaaccagcuguagucagcauuacauacaaca			
....uuugguccccuucaaccagcuguC...................	2	1	Mm2
....uuugguccccuucaaccagcuguU...................	1266	1	Mm2
....uuugguccccuucaaccagcuguG...................	3	1	Mm2
....Cuugguccccuucaaccagcugua...................	1	1	Mm2
....uuugguccccuucaaccagcugua...................	58	0	Mm2
....uuugguccccuucaaccagcuguag..................	1	0	Mm2
....uuugguccccuucaaccagcuguaU..................	17	1	Mm2
....uuugguccccuucaaccagcuguaA..................	1	1	Mm2
....uugguccccuucaaccag........................	5	0	Mm2
....uugguccccuucaaccaU........................	1	1	Mm2
....uugguccccuucaaccagU.......................	3	1	Mm2
....uugguccccuucaaccagc.......................	93	0	Mm2
....uuggAccccuucaaccagc.......................	1	1	Mm2
....uuCguccccuucaaccagc.......................	1	1	Mm2
....uuAguccccuucaaccagc.......................	1	1	Mm2
....uugguccccuucaaccagA.......................	1	1	Mm2
....uugguccccuucaaccCgcu......................	1	1	Mm2
....uugguccccuAcaaccagcu......................	1	1	Mm2
....uugguccccuucaaccaAcu......................	2	1	Mm2
....uAgguccccuucaaccagcu......................	1	1	Mm2
....uugguccccuucaaccUgcu......................	1	1	Mm2
....uugguccccuucaaccaUcu......................	1	1	Mm2
....uugguccccuucaaccagcu......................	307	0	Mm2
....uugguccccuucaaccagcA......................	3	1	Mm2
....uugguccccuucaaccaCcu......................	2	1	Mm2
....uugguccccuucaaccagcG......................	8	1	Mm2
....uuggucccUuucaaccagcu......................	1	1	Mm2
....uugguccccuGcaaccagcu......................	1	1	Mm2
....uugguccccuucaaccGgcu......................	1	1	Mm2
....uugguccccuuUaaccagcug.....................	1	1	Mm2
....uuggCccccuucaaccagcug.....................	1	1	Mm2
....uugguccccuucaaccCgcug.....................	3	1	Mm2
....uugguccccuucaaccUgcug.....................	2	1	Mm2
....uugguccccuucaaccagcGg.....................	2	1	Mm2
....uugguccUcuucaaccagcug.....................	1	1	Mm2
....uugguccccuucaaccagcug.....................	526	0	Mm2
....uugguccccuucaUccagcug.....................	1	1	Mm2
....uugguccccuucaaccGgcug.....................	1	1	Mm2
....uugguccccuucaaccagcuA.....................	11	1	Mm2
....uugguccccuucaacUagcug.....................	1	1	Mm2
....uugguccccuucaaccaUcug.....................	1	1	Mm2
....uugguccccuCcaaccagcug.....................	1	1	Mm2
....uugguccccuucaaccaAcug.....................	3	1	Mm2
....uugguccccuucaaccagcuU.....................	30	1	Mm2
....uugguccccuAcaaccagcug.....................	1	1	Mm2
....uugguccccuucaaccaCcug.....................	1	1	Mm2
....uugguccccuuAaaccagcugu....................	3	1	Mm2
....uuggCccccuucaaccagcugu....................	13	1	Mm2
....uugguUcccuucaaccagcugu....................	6	1	Mm2
....uugguccccuucaaccUgcugu....................	37	1	Mm2
....uugguccccuucCaccagcugu....................	1	1	Mm2
....uuCguccccuucaaccagcugu....................	1	1	Mm2
....uuggGccccuucaaccagcugu....................	3	1	Mm2
....uugguccccuGcaaccagcugu....................	43	1	Mm2
....uugguccccuucaaccaAcugu....................	84	1	Mm2
....Augguccccuucaaccagcugu....................	3	1	Mm2
....uugguccccuucaaccaUcugu....................	94	1	Mm2
....uugguccccuucaaccagcugC....................	23	1	Mm2
....Cugguccccuucaaccagcugu....................	4	1	Mm2
....uugguNcccuucaaccagcugu....................	1	1	Mm2
....uugguccccuucaaccagcugG....................	71	1	Mm2
....uugguccccuucaaccagcuAu....................	3	1	Mm2
....uugguccccuucaacUagcugu....................	63	1	Mm2
....uugguccccuucaaccagUugu....................	8	1	Mm2
....uugguccccuucUaccagcugu....................	5	1	Mm2
....uugguccccCucaaccagcugu....................	5	1	Mm2
....Nugguccccuucaaccagcugu....................	14	1	Mm2
....uugguccccuAcaaccagcugu....................	30	1	Mm2
....uugguccccuucaaccagcAgu....................	3	1	Mm2
....uuggucUccuucaaccagcugu....................	11	1	Mm2

```
                              Star                                      Mature
aggaagcaaggaaaaaugcgauuucuguagcugguugacaucgggccaaauugucuguauugacaugcauuugguccccuucaaccagcuguagucagcauuacauacaaca
......................................................................uAgguccccuucaaccagcugu....................    12      1      Mm2
......................................................................uuUguccccuucaaccagcugu....................    9       1      Mm2
......................................................................uugguccccuucaaGcagcugu....................    2       1      Mm2
......................................................................uuggucGccuucaaccagcugu....................    1       1      Mm2
......................................................................uugAuccccuucaaccagcugu....................    2       1      Mm2
......................................................................uugguccccuucaaccagcuCu....................    2       1      Mm2
......................................................................uugguccccuuGaaccagcugu....................    1       1      Mm2
......................................................................uCgguccccuucaaccagcugu....................    4       1      Mm2
......................................................................uugguccccuucaaccagcCgu....................    1       1      Mm2
......................................................................uugguccccuucaCccagcugu....................    1       1      Mm2
......................................................................uugguccccuucGaccagcugu....................    3       1      Mm2
......................................................................uuAguccccuucaaccagcugu....................    6       1      Mm2
......................................................................uugguccccuucaaUcagcugu....................    25      1      Mm2
......................................................................uugguccccuuUaaccagcugu....................    8       1      Mm2
......................................................................uugguccccuucaaccGgcugu....................    20      1      Mm2
......................................................................uugguccUcuucaaccagcugu....................    3       1      Mm2
......................................................................uugguccccuucaaccCgcugu....................    54      1      Mm2
......................................................................uugguccccuucaaccagNugu....................    1       1      Mm2
......................................................................uuggucccUuucaaccagcugu....................    15      1      Mm2
......................................................................uugguccccuucaacAagcugu....................    1       1      Mm2
......................................................................uugguccccuucaaccagAugu....................    2       1      Mm2
......................................................................uugguccccuucaGccagcugu....................    3       1      Mm2
......................................................................uugguAcccuucaaccagcugu....................    3       1      Mm2
......................................................................uugguccccuucaaccaCcugu....................    119     1      Mm2
......................................................................uuggAccccuucaaccagcugu....................    3       1      Mm2
......................................................................uugguccccuucaaccagcGgu....................    1       1      Mm2
......................................................................uGgguccccuucaaccagcugu....................    2       1      Mm2
......................................................................uugguccccuucaaccagcuUu....................    9       1      Mm2
......................................................................uugguccccuucaaccagGugu....................    1       1      Mm2
......................................................................uugguccccuucaaccagcugA....................    35      1      Mm2
......................................................................uuggucccAuucaaccagcugu....................    4       1      Mm2
......................................................................uugguccccuucaUccagcugu....................    4       1      Mm2
......................................................................uugguGcccuucaaccagcugu....................    2       1      Mm2
......................................................................uugguccAcuucaaccagcugu....................    1       1      Mm2
......................................................................uugguccccuCcaaccagcugu....................    18      1      Mm2
......................................................................uugguccccuucaaccagcugu....................    22139   0      Mm2
......................................................................uugguccccuucaaccagcugCa...................    1       1      Mm2
......................................................................uugguccccuucaaccagcugua...................    966     0      Mm2
......................................................................uugguccccuucaacUagcugua...................    2       1      Mm2
......................................................................uugguccccuucaaccagcuguG...................    23      1      Mm2
......................................................................uugCuccccuucaaccagcugua...................    1       1      Mm2
......................................................................uugguccccuucaaccagcuguC...................    12      1      Mm2
......................................................................uugguccccuAcaaccagcugua...................    1       1      Mm2
......................................................................uugguccccuucaaccCgcugua...................    3       1      Mm2
......................................................................uugguccccuucaaccaAcugua...................    3       1      Mm2
......................................................................uuAguccccuucaaccagcugua...................    3       1      Mm2
......................................................................uugguccccuucaaccUgcugua...................    2       1      Mm2
......................................................................uugguccccuucaaccagcugAa...................    1       1      Mm2
......................................................................uugguccccuuUaaccagcugua...................    1       1      Mm2
......................................................................uuggAccccuucaaccagcugua...................    1       1      Mm2
......................................................................uugguccccuucaaccagcCgua...................    1       1      Mm2
......................................................................uugguccccuucaaccGgcugua...................    3       1      Mm2
......................................................................uuggucccUuucaaccagcugua...................    3       1      Mm2
......................................................................uugguccccuucaaccaCcugua...................    3       1      Mm2
......................................................................uugguccccuucaaccagcuguU...................    15619   1      Mm2
......................................................................Nugguccccuucaaccagcugua...................    1       1      Mm2
......................................................................uugguccccuucaaccaUcugua...................    3       1      Mm2
......................................................................uugguNcccuucaaccagcugua...................    1       1      Mm2
......................................................................uugguccccuucaaccagcuguaA..................    193     1      Mm2
......................................................................uugguccccuucaaccagcuguag..................    9       0      Mm2
......................................................................uugguccccuucaaccagcuguUg..................    13      1      Mm2
......................................................................uugguccccuucaaccagcuguaU..................    427     1      Mm2
......................................................................uugguccccuucaaccagcuguaUu.................    22      1      Mm2
......................................................................uugguccccuucaaccagcuguaCu.................    2       1      Mm2
......................................................................uugguccccuucaaccagcuguaAu.................    2       1      Mm2
.......................................................................uUguccccuucaaccagcugu....................    1       1      Mm2
.......................................................................ugguccccuucaaccagcugu....................    7       0      Mm2
.......................................................................ugguccccuucaaccagcuguU...................    7       1      Mm2
........................................................................gguccccuucaaccagcugu....................    2       0      Mm2
.........................................................................guccccuucaaccagcug.....................    1       0      Mm2
```

```
                            Star                                         Mature
aggaagcaaggaaaaaugcgauuucuguagcugguugacaucgggccaaauugucuguauugacaugcauuugguccccuucaaccagcuguagucagcauuacauacaaca
.........................................................................guccccuucaaccagcugu....................   1      0   Mm2

............................agcugguugacaucgggccaaau.............................................................   15     0   Fm2
............................agcugguugacaucgggccaaauugucuguauugacaugcauuu........................................   1      0   Fm2
.............................gcugguugacaucgggccaaauugucuguauugacaugU............................................   4      1   Fm2
...................................................................cauuugguccccuucaaGcagc.......................   1      1   Fm2
...................................................................cauuugguccccuucaaccaUc.......................   1      1   Fm2
...................................................................cauuugguccAcuucaaccagc.......................   1      1   Fm2
...................................................................cauuugguccccuucaaccagc.......................   92     0   Fm2
...................................................................cauuugguccUcuucaaccagc.......................   1      1   Fm2
...................................................................cauuugguccccuucaaccagU.......................   5      1   Fm2
......................................................................uugguccccuucaaccagc.......................   12     0   Fm2
......................................................................uugguccccuucaaccGgcu......................   1      1   Fm2
......................................................................uugguccccuucaaccagcG......................   5      1   Fm2
......................................................................uugguccccuucaaccagcu......................   129    0   Fm2
......................................................................uugguccccuucaaccCgcu......................   1      1   Fm2
......................................................................uugguccccAucaaccagcu......................   2      1   Fm2
......................................................................uugguccccuucaacAagcu......................   1      1   Fm2
......................................................................uugguccccuucaacAagcug.....................   1      1   Fm2
......................................................................uugguccccCucaaccagcug.....................   1      1   Fm2
......................................................................uugguccccuucaaccagcGg.....................   5      1   Fm2
......................................................................uugguccccuucaacUagcug.....................   2      1   Fm2
......................................................................uugguccccGucaaccagcug.....................   1      1   Fm2
......................................................................uugguccccuucaaccagcuA.....................   1      1   Fm2
......................................................................uugguccccuucaaccGgcug.....................   2      1   Fm2
......................................................................uugguccccuucaaccagcug.....................   287    0   Fm2
......................................................................uugguccccuucaaccUgcug.....................   2      1   Fm2
......................................................................uugguccccuucaaccagcugA....................   6      1   Fm2
......................................................................uugguccccuucaaccagcGgu....................   1      1   Fm2
......................................................................uugguccccuucaaAcagcugu....................   1      1   Fm2
......................................................................uuggucGccuucaaccagcugu....................   2      1   Fm2
......................................................................uuggucUccuucaaccagcugu....................   2      1   Fm2
......................................................................uuUguccccuucaaccagcugu....................   1      1   Fm2
......................................................................uugUuccccuucaaccagcugu....................   1      1   Fm2
......................................................................uugguccccuAcaaccagcugu....................   1      1   Fm2
......................................................................uugguccccuCcaaccagcugu....................   1      1   Fm2
......................................................................uugguccccuucaaccCgcugu....................   9      1   Fm2
......................................................................uugguUcccuucaaccagcugu....................   1      1   Fm2
......................................................................uuAguccccuucaaccagcugu....................   1      1   Fm2
......................................................................uugguccccuucaacUagcugu....................   4      1   Fm2
......................................................................uugguccccCucaaccagcugu....................   2      1   Fm2
......................................................................uuggucAccuucaaccagcugu....................   1      1   Fm2
......................................................................uugguccccuucaacAagcugu....................   9      1   Fm2
......................................................................uugguccAcuucaaccagcugu....................   1      1   Fm2
......................................................................uugguccccAucaaccagcugu....................   4      1   Fm2
......................................................................uugguccccuucaaccagcugG....................   11     1   Fm2
......................................................................uugguccccuucaaccUgcugu....................   6      1   Fm2
......................................................................uuggucccAuucaaccagcugu....................   1      1   Fm2
......................................................................uugguccccuucaacGagcugu....................   2      1   Fm2
......................................................................Nugguccccuucaaccagcugu....................   1      1   Fm2
......................................................................uugguccccuucaaccGgcugu....................   5      1   Fm2
......................................................................uugguccccuucaaccagcugu....................   2070   0   Fm2
......................................................................uugguccGcuucaaccagcugu....................   1      1   Fm2
......................................................................Cugguccccuucaaccagcugu....................   5      1   Fm2
......................................................................uugguccccuucaaccagcuguG...................   1      1   Fm2
......................................................................uugguccccuucaaccagcuguU...................   500    1   Fm2
......................................................................uugguccccuucaaccagcugua...................   68     0   Fm2
......................................................................uugguccccuucaaccUgcugua...................   1      1   Fm2
......................................................................uugguccccuucaaccagcuguaU..................   11     1   Fm2
.......................................................................ugguccccuucaaccag........................   5      0   Fm2
.......................................................................uggCccccuucaaccagc.......................   1      1   Fm2
.......................................................................ugguccccuucaaccagc.......................   93     0   Fm2
.......................................................................ugguccccuCcaaccagc.......................   1      1   Fm2
.......................................................................ugguccccuucaaccaCc.......................   1      1   Fm2
.......................................................................ugguccccuCcaaccagcu......................   1      1   Fm2
.......................................................................ugguccccuucaaccaAcu......................   5      1   Fm2
.......................................................................ugguccccuucaaccaCcu......................   2      1   Fm2
.......................................................................uggCccccuucaaccagcu......................   1      1   Fm2
.......................................................................ugguccccuucaacUagcu......................   3      1   Fm2
.......................................................................ugguccccuucaaccagcu......................   499    0   Fm2
```

Star Mature

```
aggaagcaaggaaaaaugcgauuucuguagcugguugacaucgggccaaauugucuguauugacaugcauuugguccccuucaaccagcuguagucagcauuacauacaaca
......................................................................uugguccccuucaacAagcu......................   1      1   Fm2
......................................................................uugguUcccuucaaccagcu......................   1      1   Fm2
......................................................................uugguccccuucaaccagcC......................   1      1   Fm2
......................................................................uuggucUccuucaaccagcu......................   1      1   Fm2
......................................................................Nugguccccuucaaccagcu......................   1      1   Fm2
......................................................................Augguccccuucaaccagcu......................   1      1   Fm2
......................................................................uugguccccuucaaccCgcu......................   1      1   Fm2
......................................................................uugguccccuucaaccagcG......................   15     1   Fm2
......................................................................uugguccccuuGaaccagcu......................   1      1   Fm2
......................................................................uugAuccccuucaaccagcu......................   1      1   Fm2
......................................................................uugguccccuucaaccaUcu......................   1      1   Fm2
......................................................................uugguccccuucaaccUgcu......................   1      1   Fm2
......................................................................uugguccccuAcaaccagcu......................   1      1   Fm2
......................................................................uugguccccuucaaccagcuU.....................   25     1   Fm2
......................................................................uugguccccuucaaccagcuA.....................   6      1   Fm2
......................................................................uugguccccuucaaccaAcug.....................   2      1   Fm2
......................................................................uugguccccuGcaaccagcug.....................   2      1   Fm2
......................................................................uugguccccuucaaccagcGg.....................   4      1   Fm2
......................................................................uugguccccuucaaccagcug.....................   421    0   Fm2
......................................................................uuggucUccuucaaccagcug.....................   1      1   Fm2
......................................................................uugguccUcuucaaccagcugu....................   1      1   Fm2
......................................................................uuggGccccuucaaccagcugu....................   3      1   Fm2
......................................................................uugguccccuucaaccagcCgu....................   1      1   Fm2
......................................................................uugguccccuucaaccaUcugu....................   57     1   Fm2
......................................................................uugguNcccuucaaccagcugu....................   2      1   Fm2
......................................................................uugguccccAucaaccagcugu....................   2      1   Fm2
......................................................................uugguAcccuucaaccagcugu....................   1      1   Fm2
......................................................................uuggucGccuucaaccagcugu....................   2      1   Fm2
......................................................................uugguccccuucaaccagcuUu....................   10     1   Fm2
......................................................................uugguccccuuUaaccagcugu....................   1      1   Fm2
......................................................................uugguccccuucaaccagcuAu....................   2      1   Fm2
......................................................................uugguccccuuAaaccagcugu....................   1      1   Fm2
......................................................................uAgguccccuucaaccagcugu....................   4      1   Fm2
......................................................................uugguccccuucaaUcagcugu....................   7      1   Fm2
......................................................................uuggucUccuucaaccagcugu....................   4      1   Fm2
......................................................................Augguccccuucaaccagcugu....................   1      1   Fm2
......................................................................uugguccccuucaaccagcugC....................   3      1   Fm2
......................................................................uugguccccuucaaccagcugA....................   64     1   Fm2
......................................................................uugguccccuucUaccagcugu....................   1      1   Fm2
......................................................................uugguccccuucaaccagcugu....................   12690  0   Fm2
......................................................................uugguccccuucaaccaAcugu....................   48     1   Fm2
......................................................................uugguccccuucaaccagcAgu....................   1      1   Fm2
......................................................................uugguccAcuucaaccagcugu....................   3      1   Fm2
......................................................................uugguccccuucaacGagcugu....................   1      1   Fm2
......................................................................uugguccccuucaaccaCcugu....................   65     1   Fm2
......................................................................uugguccccuucaaccUgcugu....................   30     1   Fm2
......................................................................uugUuccccuucaaccagcugu....................   2      1   Fm2
......................................................................uugguccccuucaCccagcugu....................   2      1   Fm2
......................................................................uCgguccccuucaaccagcugu....................   3      1   Fm2
......................................................................uuAguccccuucaaccagcugu....................   5      1   Fm2
......................................................................uugguccccuucaGccagcugu....................   1      1   Fm2
......................................................................uugguccccuAcaaccagcugu....................   22     1   Fm2
......................................................................uugguccccuucaaccGgcugu....................   13     1   Fm2
......................................................................uugguUcccuucaaccagcugu....................   12     1   Fm2
......................................................................uugguccccuCcaaccagcugu....................   13     1   Fm2
......................................................................uugguccccuucaaccagcugG....................   53     1   Fm2
......................................................................uuCguccccuucaaccagcugu....................   4      1   Fm2
......................................................................uugAuccccuucaaccagcugu....................   7      1   Fm2
......................................................................uugguccccuucaacUagcugu....................   118    1   Fm2
......................................................................uugguccccuucaaccCgcugu....................   28     1   Fm2
......................................................................uuggucccUuucaaccagcugu....................   9      1   Fm2
......................................................................uugguccccuucaaAcagcugu....................   1      1   Fm2
......................................................................uuggCccccuucaaccagcugu....................   5      1   Fm2
......................................................................uugguccccuuGaaccagcugu....................   1      1   Fm2
......................................................................uugCuccccuucaaccagcugu....................   2      1   Fm2
......................................................................uGgguccccuucaaccagcugu....................   1      1   Fm2
......................................................................Gugguccccuucaaccagcugu....................   1      1   Fm2
......................................................................uugguccccuGcaaccagcugu....................   18     1   Fm2
......................................................................Nugguccccuucaaccagcugu....................   7      1   Fm2
......................................................................uuggAccccuucaaccagcugu....................   5      1   Fm2
```

```
                            Star                                         Mature
aggaagcaaggaaaaaugcgauuucuguagcugguugacaucgggccaaauugucuguauugacaugcauuugguccccuucaaccagcuguagucagcauuacauacaaca
......................................................................uugguccccuucaUccagcugu....................   3       1   Fm2
......................................................................uugguccccuucaaccagAugu....................   1       1   Fm2
......................................................................uugguccccuucGaccagcugu....................   1       1   Fm2
......................................................................uuUguccccuucaaccagcugu....................   8       1   Fm2
......................................................................uugguGcccuucaaccagcugu....................   1       1   Fm2
......................................................................uugguccccuucaaccagcuguU...................   14061   1   Fm2
......................................................................uugguccUcuucaaccagcugua...................   1       1   Fm2
......................................................................uugguccccuucaaccCgcugua...................   3       1   Fm2
......................................................................uuggCccccuucaaccagcugua...................   1       1   Fm2
......................................................................uugguccccuGcaaccagcugua...................   2       1   Fm2
......................................................................uugguccccuucaaccagcugua...................   1062    0   Fm2
......................................................................uuggucUccuucaaccagcugua...................   1       1   Fm2
......................................................................uugguccccuAcaaccagcugua...................   2       1   Fm2
......................................................................uugguccccuucaaccagcuguC...................   22      1   Fm2
......................................................................uugguccccuucaaccaUcugua...................   1       1   Fm2
......................................................................uugguccccuucaaccaCcugua...................   5       1   Fm2
......................................................................uugguccccuucaaccagcuguG...................   22      1   Fm2
......................................................................uugguccccuucaaccaAcugua...................   4       1   Fm2
......................................................................uCgguccccuucaaccagcugua...................   1       1   Fm2
......................................................................uugguccccuucaaccUgcugua...................   3       1   Fm2
......................................................................uugguccccuucaaccagcuguaA..................   98      1   Fm2
......................................................................uugguccccuucaaccagcuguUg..................   21      1   Fm2
......................................................................uugguccccuucaaccagcuguaU..................   153     1   Fm2
......................................................................uugguccccuucaaccagcuguaUu.................   35      1   Fm2
......................................................................uugguccccuucaaccagcuguaAu.................   1       1   Fm2
.......................................................................ugguccccuucaaccagcugu....................   6       0   Fm2
.......................................................................ugguccccuucaaccagcugua...................   1       0   Fm2
........................................................................Uguccccuucaaccagcugu....................   1       1   Fm2

............................agcugguugacaucgggccaaa..............................................................   2       0   Fm1
............................agcugguugacaucgggGcaaau.............................................................   1       1   Fm1
............................agcugguugacaucgggccaaau.............................................................   28      0   Fm1
............................agcugguugacaucgggAcaaau.............................................................   1       1   Fm1
...................................................................cauuugguccccuucaaccagc.......................   35      0   Fm1
...................................................................cauuugguccccuucaaccagcu......................   4       0   Fm1
....................................................................auuugguccccuucaaccagcu......................   1       0   Fm1
....................................................................Cuuugguccccuucaaccagcugu....................   1       1   Fm1
.....................................................................uuugguccccuucaacca.........................   3       0   Fm1
.....................................................................uuugguccccuucaaccag........................   6       0   Fm1
.....................................................................uuugguccccuucaacUagc.......................   1       1   Fm1
.....................................................................uuuggucccUuucaaccagc.......................   1       1   Fm1
.....................................................................uuugguccccuucaaccUgc.......................   1       1   Fm1
.....................................................................uuuAguccccuucaaccagc.......................   1       1   Fm1
.....................................................................uuugguccccuucaaccagc.......................   156     0   Fm1
.....................................................................uuugguccccuucaaccagU.......................   3       1   Fm1
.....................................................................uuugguccccuucaaccCgcu......................   4       1   Fm1
.....................................................................uuugguccccuucaaccagcA......................   1       1   Fm1
.....................................................................uuugguccccuucaaccagcu......................   876     0   Fm1
.....................................................................uuugguccccuucaaccagcG......................   18      1   Fm1
.....................................................................uuugguccccuucaacUagcu......................   3       1   Fm1
.....................................................................uuugguccccuucaacAagcu......................   3       1   Fm1
.....................................................................Nuugguccccuucaaccagcu......................   1       1   Fm1
.....................................................................uuugguccccuucaaccGgcu......................   1       1   Fm1
.....................................................................uuugguccccuucaaccUgcu......................   4       1   Fm1
.....................................................................uuuCguccccuucaaccagcu......................   1       1   Fm1
.....................................................................uuugguccccuucaaccagcC......................   1       1   Fm1
.....................................................................uAugguccccuucaaccagcu......................   2       1   Fm1
.....................................................................uuugguccccAucaaccagcu......................   4       1   Fm1
.....................................................................uuugguccccuucaaccagcuU.....................   23      1   Fm1
.....................................................................uuugguccccuucaaccaCcug.....................   1       1   Fm1
.....................................................................uuugguccccuucaaccagcuA.....................   11      1   Fm1
.....................................................................uuugguccccuucaaccagcuC.....................   1       1   Fm1
.....................................................................Nuugguccccuucaaccagcug.....................   1       1   Fm1
.....................................................................uuugguccccuucaaccagcCg.....................   1       1   Fm1
.....................................................................uuugguccccuucaacUagcug.....................   4       1   Fm1
.....................................................................uuugguccccuucaaccCgcug.....................   6       1   Fm1
.....................................................................uuugguccAcuucaaccagcug.....................   1       1   Fm1
.....................................................................uuugguccccAucaaccagcug.....................   1       1   Fm1
.....................................................................uuuggCccccuucaaccagcug.....................   1       1   Fm1
.....................................................................uuugguccccuucaacAagcug.....................   7       1   Fm1
```

Star **Mature**

aggaagcaaggaaaaaugcgauuucuguagcugguugacaucgggccaaauugucuguauugacaugcauuugguccccuucaaccagcuguagucagcauuacauacaaca			
....uuugguccccuucaaUcagcug.....................	3	1	Fm1
....uuugguccccuucaacGagcug.....................	1	1	Fm1
....uuugguccccuucaaccUgcug.....................	7	1	Fm1
....uuugguccccuucaaccagcug.....................	1328	0	Fm1
....uuugguccccuucaaccagcGg.....................	18	1	Fm1
....uuuggNccccuucaaccagcug.....................	1	1	Fm1
....uuuggucccUuucaaccagcug.....................	5	1	Fm1
....uuugguccUcuucaaccagcug.....................	7	1	Fm1
....uuugguccccuucaaccGgcug.....................	10	1	Fm1
....uuugguccccGucaaccagcug.....................	6	1	Fm1
....uuugguccccuucaGccagcugu....................	2	1	Fm1
....uuuggGccccuucaaccagcugu....................	1	1	Fm1
....uuugguccccuucaaccUgcugu....................	28	1	Fm1
....uuugguccccuAcaaccagcugu....................	1	1	Fm1
....uuugguccccuucaaccagcuUu....................	3	1	Fm1
....uuugguUcccuucaaccagcugu....................	10	1	Fm1
....uuugAuccccuucaaccagcugu....................	1	1	Fm1
....uuugguccccAucaaccagcugu....................	10	1	Fm1
....uuugguGcccuucaaccagcugu....................	1	1	Fm1
....uuugCuccccuucaaccagcugu....................	1	1	Fm1
....uuugguccGcuucaaccagcugu....................	2	1	Fm1
....uuugguccccuuUaaccagcugu....................	7	1	Fm1
....uuugguccccuucaaccagcugA....................	20	1	Fm1
....uuGgguccccuucaaccagcugu....................	2	1	Fm1
....uuCgguccccuucaaccagcugu....................	1	1	Fm1
....uuugguccccuucaacUagcugu....................	25	1	Fm1
....uuugguccccuucaaUcagcugu....................	3	1	Fm1
....uuugguccccuucaaccagcugu....................	6622	0	Fm1
....uuugguccccuucaacAagcugu....................	24	1	Fm1
....uuugguccccuucaaccGgcugu....................	23	1	Fm1
....uuuggNccccuucaaccagcugu....................	2	1	Fm1
....Cuugguccccuucaaccagcugu....................	9	1	Fm1
....uuuggAccccuucaaccagcugu....................	3	1	Fm1
....uuugguAcccuucaaccagcugu....................	1	1	Fm1
....uuuUguccccuucaaccagcugu....................	1	1	Fm1
....uuugguccccuucCaccagcugu....................	1	1	Fm1
....uuuggucUccuucaaccagcugu....................	6	1	Fm1
....uuuggucAccuucaaccagcugu....................	1	1	Fm1
....Nuugguccccuucaaccagcugu....................	2	1	Fm1
....uuugguccccuucaaccaUcugu....................	1	1	Fm1
....uuugguccccuucaaccaAcugu....................	1	1	Fm1
....uuuggucccUuucaaccagcugu....................	19	1	Fm1
....uuugguccccuucaaccagUugu....................	8	1	Fm1
....uCugguccccuucaaccagcugu....................	2	1	Fm1
....uuugguccccCucaaccagcugu....................	4	1	Fm1
....uuugguccccuucaaccagcugG....................	41	1	Fm1
....uuugguccccuucaUccagcugu....................	2	1	Fm1
....uuuAguccccuucaaccagcugu....................	1	1	Fm1
....uuugguccUcuucaaccagcugu....................	7	1	Fm1
....uuugguccccuucaaccCgcugu....................	22	1	Fm1
....uAugguccccuucaaccagcugu....................	2	1	Fm1
....uuugguccccuucaacGagcugu....................	5	1	Fm1
....uuugguccccuucGaccagcugu....................	1	1	Fm1
....uuugguccccGucaaccagcugu....................	7	1	Fm1
....uuugguccccuucaaccagcugC....................	24	1	Fm1
....uuugguccccuucaaccagcuguU...................	2234	1	Fm1
....uuugguccccuucaaccagcugCa...................	8	1	Fm1
....uuugguccccuucaaccagcugua...................	149	0	Fm1
....uuugguccccuucaacAagcugua...................	2	1	Fm1
....uuugguccccuucaaccagcuguG...................	2	1	Fm1
....uuugguccccuucaaccagcuguaU..................	21	1	Fm1
....uugguccccuucaaccaU........................	2	1	Fm1
....uugguccccuucaaccaA........................	1	1	Fm1
....uugguccccuucaaccag........................	9	0	Fm1
....uugguccccuucaaccGg........................	1	1	Fm1
....uugguccccuucaaccagG.......................	1	1	Fm1
....uugguUcccuucaaccagc.......................	1	1	Fm1
....uugguccccuGcaaccagc.......................	1	1	Fm1
....uugguccccuucaaccagU.......................	11	1	Fm1
....uugguccccuucaaccaAc.......................	5	1	Fm1

```
                            Star                                       Mature
aggaagcaaggaaaaaugcgauuucuguagcugguugacaucgggccaaauugucuguauugacaugcauuugguccccuucaaccagcuguagucagcauuacauacaaca
......................................................................uugguccccuucaaccagc.......................	849	0	Fm1
......................................................................uugguccccuAcaaccagc.......................	3	1	Fm1
......................................................................Nugguccccuucaaccagc.......................	1	1	Fm1
......................................................................uugguccccuucaaccagA.......................	11	1	Fm1
......................................................................uugguAcccuucaaccagc.......................	1	1	Fm1
......................................................................uugguccccuucaaccaUc.......................	3	1	Fm1
......................................................................uGgguccccuucaaccagc.......................	1	1	Fm1
......................................................................uugguccccuCcaaccagc.......................	1	1	Fm1
......................................................................uugguccccuucaaccCgc.......................	4	1	Fm1
......................................................................uugguccccuucaaccUgc.......................	3	1	Fm1
......................................................................uugguccccuucaaccaCc.......................	6	1	Fm1
......................................................................Cugguccccuucaaccagc.......................	1	1	Fm1
......................................................................uugguccccuucaaccGgcu......................	3	1	Fm1
......................................................................uugguccccuucaaccaUcu......................	10	1	Fm1
......................................................................uugguccccuucaaccaAcu......................	13	1	Fm1
......................................................................uuCguccccuucaaccagcu......................	2	1	Fm1
......................................................................uuggGccccuucaaccagcu......................	1	1	Fm1
......................................................................uugguccccuucaaccUgcu......................	1	1	Fm1
......................................................................uugguccccuucaaccNgcu......................	1	1	Fm1
......................................................................uugguccccuucaacUagcu......................	4	1	Fm1
......................................................................uugguccccuucaaccagcG......................	76	1	Fm1
......................................................................uCgguccccuucaaccagcu......................	1	1	Fm1
......................................................................uugguccccuucaaccagcA......................	11	1	Fm1
......................................................................uugguNcccuucaaccagcu......................	1	1	Fm1
......................................................................uuAguccccuucaaccagcu......................	1	1	Fm1
......................................................................uugguccccuucaaccagcu......................	2454	0	Fm1
......................................................................uugguGcccuucaaccagcu......................	2	1	Fm1
......................................................................uAgguccccuucaaccagcu......................	1	1	Fm1
......................................................................uugguccccuuUaaccagcu......................	1	1	Fm1
......................................................................Nugguccccuucaaccagcu......................	1	1	Fm1
......................................................................uugguUcccuucaaccagcu......................	2	1	Fm1
......................................................................uugguccccuGcaaccagcu......................	1	1	Fm1
......................................................................uugguccccuCcaaccagcu......................	2	1	Fm1
......................................................................uuggucccUuucaaccagcu......................	4	1	Fm1
......................................................................uugguccccuAcaaccagcu......................	4	1	Fm1
......................................................................uuUguccccuucaaccagcu......................	2	1	Fm1
......................................................................uugguccccuucaaccCgcu......................	8	1	Fm1
......................................................................Augguccccuucaaccagcu......................	1	1	Fm1
......................................................................uugguccccuucaaccaCcu......................	10	1	Fm1
......................................................................uuggucUccuucaaccagcu......................	1	1	Fm1
......................................................................uuggucccUuucaaccagcug.....................	3	1	Fm1
......................................................................uuAguccccuucaaccagcug.....................	1	1	Fm1
......................................................................uugguccccuucaaccagcuU.....................	86	1	Fm1
......................................................................uugguccccuuUaaccagcug.....................	5	1	Fm1
......................................................................uugguccccuucaaccCgcug.....................	7	1	Fm1
......................................................................uugguccccuucaGccagcug.....................	1	1	Fm1
......................................................................uugguccccuucaaccaAcug.....................	6	1	Fm1
......................................................................uugguccccuGcaaccagcug.....................	2	1	Fm1
......................................................................uugguccccuucaaccagcuA.....................	17	1	Fm1
......................................................................uugguccccuCcaaccagcug.....................	2	1	Fm1
......................................................................uugguccccuucaaccUgcug.....................	3	1	Fm1
......................................................................uugguccccuucaaccaCcug.....................	6	1	Fm1
......................................................................uugguccccuucaUccagcug.....................	1	1	Fm1
......................................................................uugguccccuucaaccaUcug.....................	16	1	Fm1
......................................................................uugguccccuucaaccagcug.....................	1517	0	Fm1
......................................................................Cugguccccuucaaccagcug.....................	1	1	Fm1
......................................................................uugguccccuucaaccagcGg.....................	17	1	Fm1
......................................................................uuggAccccuucaaccagcug.....................	1	1	Fm1
......................................................................uugguccccuucaacGagcug.....................	1	1	Fm1
......................................................................uugguccccuucaaccGgcug.....................	1	1	Fm1
......................................................................uuggCccccuucaaccagcug.....................	1	1	Fm1
......................................................................uugguccccuAcaaccagcug.....................	1	1	Fm1
......................................................................uugUuccccuucaaccagcugu....................	3	1	Fm1
......................................................................Cugguccccuucaaccagcugu....................	10	1	Fm1
......................................................................uugguccccuucaUccagcugu....................	11	1	Fm1
......................................................................uuggAccccuucaaccagcugu....................	3	1	Fm1
......................................................................Gugguccccuucaaccagcugu....................	2	1	Fm1
......................................................................uugguccccuucaaccagGugu....................	2	1	Fm1
......................................................................uugguccAcuucaaccagcugu....................	4	1	Fm1
......................................................................uugguccccuAcaaccagcugu....................	75	1	Fm1
```

Star Mature

```
aggaagcaaggaaaaaugcgauuucuguagcugguugacaucgggccaaauugucuguauugacaugcauuugguccccuucaaccagcuguagucagcauuacauacaaca
......................................................................uugguUcccuucaaccagcugu....................  16     1  Fm1
......................................................................uugAuccccuucaaccagcugu....................  15     1  Fm1
......................................................................uugguccccuucaaccagcAgu....................  1      1  Fm1
......................................................................uuggucccUuucaaccagcugu....................  35     1  Fm1
......................................................................uugguccccuucaaccGgcugu....................  28     1  Fm1
......................................................................uAgguccccuucaaccagcugu....................  14     1  Fm1
......................................................................uugguGcccuucaaccagcugu....................  1      1  Fm1
......................................................................uuggucccGuucaaccagcugu....................  1      1  Fm1
......................................................................uugguccccuucaaUcagcugu....................  13     1  Fm1
......................................................................uugguccccuucaaccCgcugu....................  87     1  Fm1
......................................................................uugguccccuucaacUagcugu....................  109    1  Fm1
......................................................................uugguccccCucaaccagcugu....................  17     1  Fm1
......................................................................uugguccccuucaaccUgcugu....................  67     1  Fm1
......................................................................uugguAcccuucaaccagcugu....................  4      1  Fm1
......................................................................uugguccccuucaaccagcGgu....................  2      1  Fm1
......................................................................uugguccccuucaGccagcugu....................  3      1  Fm1
......................................................................uuAguccccuucaaccagcugu....................  8      1  Fm1
......................................................................uugguccccuucaaccagcugC....................  39     1  Fm1
......................................................................uuggucGccuucaaccagcugu....................  1      1  Fm1
......................................................................uugguccccuucaaccagcuUu....................  16     1  Fm1
......................................................................uCgguccccuucaaccagcugu....................  7      1  Fm1
......................................................................uugguccccuucaaccaCcugu....................  183    1  Fm1
......................................................................uugguccccuucaCccagcugu....................  2      1  Fm1
......................................................................uugguccccuucaaccagUugu....................  20     1  Fm1
......................................................................Augguccccuucaaccagcugu....................  3      1  Fm1
......................................................................uugguccccuucaaGcagcugu....................  2      1  Fm1
......................................................................uuggucccAuucaaccagcugu....................  12     1  Fm1
......................................................................uugguccccuucaaccaUcugu....................  221    1  Fm1
......................................................................uuggucUccuucaaccagcugu....................  9      1  Fm1
......................................................................uugguccccuucaaccagcugu....................  39398  0  Fm1
......................................................................uugguccccuucaaccagcuCu....................  2      1  Fm1
......................................................................uugguccUcuucaaccagcugu....................  8      1  Fm1
......................................................................uuggGccccuucaaccagcugu....................  4      1  Fm1
......................................................................uugguccccuucaaccagcuAu....................  3      1  Fm1
......................................................................uuUguccccuucaaccagcugu....................  14     1  Fm1
......................................................................uugCuccccuucaaccagcugu....................  2      1  Fm1
......................................................................uugguccccuuAaaccagcugu....................  2      1  Fm1
......................................................................uugguccccuCcaaccagcugu....................  29     1  Fm1
......................................................................uugguccccuuUaaccagcugu....................  32     1  Fm1
......................................................................Nugguccccuucaaccagcugu....................  12     1  Fm1
......................................................................uugguccccuucaaAcagcugu....................  2      1  Fm1
......................................................................uugguccccuucaaccaAcugu....................  197    1  Fm1
......................................................................uugguccccuucCaccagcugu....................  1      1  Fm1
......................................................................uugguccccuucaaccagcugA....................  71     1  Fm1
......................................................................uuggCccccuucaaccagcugu....................  14     1  Fm1
......................................................................uugguccccuucaaccagcCgu....................  8      1  Fm1
......................................................................uugguccccuucaaccagcugG....................  196    1  Fm1
......................................................................uugguccccuucUaccagcugu....................  6      1  Fm1
......................................................................uGgguccccuucaaccagcugu....................  6      1  Fm1
......................................................................uuggucNccuucaaccagcugu....................  2      1  Fm1
......................................................................uugguccccAucaaccagcugu....................  3      1  Fm1
......................................................................uuCguccccuucaaccagcugu....................  1      1  Fm1
......................................................................uugguccccuucaaccagAugu....................  8      1  Fm1
......................................................................uuggucAccuucaaccagcugu....................  7      1  Fm1
......................................................................uugguccccuucGaccagcugu....................  6      1  Fm1
......................................................................uugguccccuucaacAagcugu....................  2      1  Fm1
......................................................................uugguccccuGcaaccagcugu....................  63     1  Fm1
......................................................................uugguccccuucaaccagcuguC...................  28     1  Fm1
......................................................................uugguccccuucaaccGgcugua...................  1      1  Fm1
......................................................................uugguccccuucaaccagcugAa...................  18     1  Fm1
......................................................................uugguccccuCcaaccagcugua...................  2      1  Fm1
......................................................................uugguccccuucaaccagcuAua...................  1      1  Fm1
......................................................................uugguccUcuucaaccagcugua...................  1      1  Fm1
......................................................................uugguccccuAcaaccagcugua...................  3      1  Fm1
......................................................................uugguccccuucaaccaAcugua...................  10     1  Fm1
......................................................................uugAuccccuucaaccagcugua...................  1      1  Fm1
......................................................................uugguccccuGcaaccagcugua...................  1      1  Fm1
......................................................................uugguccccuucaaccagcugCa...................  2      1  Fm1
......................................................................uugguccccuucaacGagcugua...................  1      1  Fm1
......................................................................uugguccccuucaaccagcugua...................  1402   0  Fm1
```

```
                            Star                                         Mature
aggaagcaaggaaaaaugcgauuucuguagcugguugacaucgggccaaauugucuguauugacaugcauuugguccccuucaaccagcuguagucagcauuacauacaaca
......................................................................uugguccccuucaaccUgcugua...................   5      1   Fm1
......................................................................uugguccccuuUaaccagcugua...................   3      1   Fm1
......................................................................uugguccccuucaaccaCcugua...................   6      1   Fm1
......................................................................uugguccccuucaaccagcuguU...................   23996  1   Fm1
......................................................................uuggCccccuucaaccagcugua...................   1      1   Fm1
......................................................................uugguccccuucaaccCgcugua...................   3      1   Fm1
......................................................................uuggAccccuucaaccagcugua...................   1      1   Fm1
......................................................................uugguccccuucaaccagAugua...................   3      1   Fm1
......................................................................uugguccccuucaaccaUcugua...................   3      1   Fm1
......................................................................uugguccccuucaaccagcuguG...................   35     1   Fm1
......................................................................uugguccccuucaaccagcuguaU..................   606    1   Fm1
......................................................................uugguccccuucaaccagcuguaA..................   235    1   Fm1
......................................................................uugguccccuucaaccagcuguaC..................   1      1   Fm1
......................................................................uugguccccuucaaccagcuguCg..................   1      1   Fm1
......................................................................uugguccccuucaaccagcuguGg..................   2      1   Fm1
......................................................................uugguccccuucaaccagcuguag..................   5      0   Fm1
......................................................................uugguccccuucaaccagcuguUg..................   37     1   Fm1
......................................................................uugguccccuucaaccagcuguaUu.................   22     1   Fm1
......................................................................uugguccccuucaaccagcuguaCu.................   3      1   Fm1
......................................................................uugguccccuucaaccagcuguaAu.................   14     1   Fm1
.......................................................................ugguccccuucaaccagcu......................   2      0   Fm1
.......................................................................ugguccccuucaaccagcugu....................   20     0   Fm1
.......................................................................ugguccccuucaaccGgcugu....................   1      1   Fm1
.......................................................................ugguccccuucaaccagcuguU...................   8      1   Fm1
........................................................................gguccccuucaaccagcu......................   1      0   Fm1
........................................................................gguccccuucaaccagcugu....................   3      0   Fm1
```
